# The Oral Tolerance as a Complex Network Phenomenon

**DOI:** 10.1371/journal.pone.0130762

**Published:** 2015-06-26

**Authors:** Pedro Jeferson Miranda, Murilo Delgobo, Giovani Favero Marino, Kátia Sabrina Paludo, Murilo da Silva Baptista, Sandro Ely de Souza Pinto

**Affiliations:** 1 Department of Physics, State University of Ponta Grossa, Ponta Grossa, Paraná, Brazil; 2 Department of Biology, State University of Ponta Grossa, Ponta Grossa, Paraná, Brazil; 3 Institute for Complex Systems and Mathematical Biology, SUPA, University of Aberdeen, Aberdeen, United Kingdom; 4 Department of Structural Biology, Molecular and Genetics, State University of Ponta Grossa, Ponta Grossa, Paraná, Brazil; Harvard Medical School, UNITED STATES

## Abstract

The phenomenon of oral tolerance refers to a local and systemic state of tolerance induced in the gut after its exposure to innocuous antigens. Recent findings have shown the interrelationship between cellular and molecular components of oral tolerance, but its representation through a network of interactions has not been investigated. Our work aims at identifying the causal relationship of each element in an oral tolerance network, and also to propose a phenomenological model that’s capable of predicting the stochastic behavior of this network when under manipulation. We compared the changes of a “healthy” network caused by “knock-outs” (KOs) in two approaches: an analytical approach by the Perron Frobenius theory; and a computational approach, which we describe within this work in order to find numerical results for the model. Both approaches have shown the most relevant immunological components for this phenomena, that happens to corroborate the empirical results from animal models. Besides explain in a intelligible fashion how the components interacts in a complex manner, we also managed to describe and quantify the importance of KOs that hasn’t been empirically tested.

## Introduction

The adaptive immune response (AIR) constitutes a remarkable characteristic of jawed vertebrate organisms, given its plasticity and specificity of systemic responses. In general, it is mostly composed of antibody secreting cells, also known as B cells, and by a set of specialized T cells, which modulates distinct functions on immune response [1]. By plasticity and specificity of AIR we mean that while it has the ability to eliminate harmful pathogens, it must also generate a tolerance to benign antigens to avoid immunological responses against self-components of the system. These tolerated antigens are composed by self-structures and environment antigens, such as food proteins obtained by diet [2–6].

Oral tolerance refers to a local and systemic state of tolerance induced in the gut associated lymphoid tissues (GALT) after its exposure to innocuous antigens, such as food proteins [3]. Antigen presenting cells (APCs) resident in intestinal lamina propria (LP) capture antigens from the lumen and migrate to mesenteric lymph nodes (mLN), where they drive T cell differentiation. Tregs generated in the mLN may return to the LP or enter bloodstream via spleen, where they promote the systemic effects of oral tolerance [4]. While immunology has made solid advances in terms of defining the genetics, molecular and cellular components involved in oral tolerance phenomena [5, 6], its representations through a network of interactions among its components and the effect of the network in the behavior of the oral tolerance has not been investigated.

As a matter of fact, oral tolerance relies on the complex interactions of immune components in a unique microenvironment (GALT) [7]. We considered a phenomenological stochastic network model, based on a random walker approach that encompasses functional responses such as the ones observed in oral tolerance. To verify the validity of our model, we individually removed (termed as KO) immunological components (i. e. silencing a vertex) and study how the dynamics of the KO’s network differs, when compared to the standard network. The results from these simulated KOs are then compared to the respective *in vivo* knockout models, for major constributors of oral tolerance induction and maintenance. Additionally, we compare the statistics coming from the numerical simulations with the invariant asymptotic probabilities obtained analytically by the adjacency matrix of the network. The analytical method is applicable when the transition matrix satisfies the Perron Frobenius theory; otherwise, the numerical approach will work for any case.

## Material and Methods

### The oral tolerance as a complex network phenomenon

To model the oral tolerance phenomenon, we approach our problem using a complex network, which represents the known immunological relations. We also seek to explain and quantify the importance of each immunological component (i. e. vertices) in terms of the global network dynamics. The first consideration taken from this approach is the association of our phenomenon as a systemic response to a stimulus. This initial stimulus unchains a process of special interactions of immunological components. The phenomenon per se should rely on how these components are related to each other and when these interactions occur. Given these premises, we propose a network which captures these interactions and a time dependent quantity associated to the diffusion of stimulation in the network. This diffusion, since we are modeling the oral tolerance, stands for the constant entrance of antigens in the GALT. We propose that this process occurs like a random walk process, since it is the simplest way to model diffusion phenomena.

### Network assembly

The interaction’s network of oral tolerance was built from reliable experimental data in the literature concerning oral tolerance induction and maintenance and adapted to our model. Immune cellular and molecular components involved in oral tolerance induction and maintenance such as Treg, cells, IL-10, CCL25 and antigens were represented as network’s vertices. The interactions, regulatory relationships and transformations among components were described as directed edges, starting from the source vertex and ending on the target vertex. The network’s vertices selection considered an initial phase of antigen entry and sampling from intestinal lumen, antigen presentation to *naïve* CD4^+^ T cells, generation of Treg cells and Treg homing and expansion in LP. In regards to the individual network’s KO, our data was compared with mice knockout models and in some cases (*e*.*g*., tight junctions) we compared with agents that modulate the system *in vivo* (*e*.*g*., zonulin) [8].

**Fig 1:**
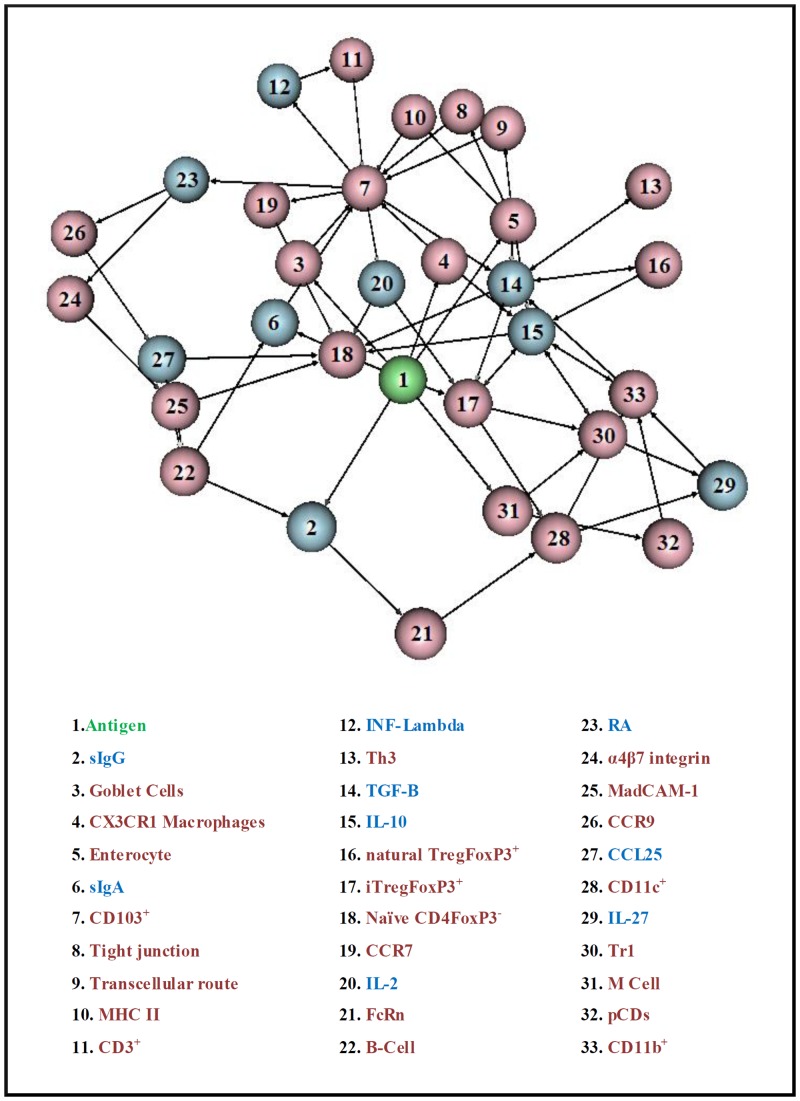
Relational immunological network comprising elements involved in the Oral Tolerance. Pink vertices (23) are related to cellular components and light blue components (9) to molecular component; the green vertex represents the source of walkers, which stands for Antigens. Its associated table below shows the immunological component related to its displayed numeric label.

On the total, we considered 9 molecular and 23 cellular components relevant to the dynamical process, plus the antigen (*i*. *e*., the starting vertex). Some foreign peptides (antigens) can resist both the low pH of the gastric fluid and proteolytic enzyme hydrolysis, reaching the small intestine lumen as large immunogenic peptides or intact proteins [9]. These antigens can be complexed to IgG and IgA in the lumen, and transported to intestinal lamina propria (LP) through neonatal Fc receptor (FcRn) and transferrin receptor (CD71) respectively [9, 10]. Enterocytes, known as intestine absorptive cells, plays a critical role in antigens capture. Small molecules (<600 Da), like degraded proteins, may pass through enterocytes’s tight junction and can fuse to MHC II compartments delivering antigens to *intestinal lamina* propria in exosomes [4, 11]. Antigens can also be transported to Peyer’s Patches through M cells also mediates oral tolerance induction [12]. A particular population of dendritic cells (DCs) residing in the LP (DCs CD103^+^) receives and loads the antigens from intestinal lumen. These cells migrate to mesenteric lymph nodes (mLN) where they induce the differentiation of naïve CD4 T cells to T regulatory cells [13]. CD103^+^ DCs present antigens via MHC II combined with transforming growth factor β (TGF-β) and retinoic acid (RA), favoring the differentiation of iTregsFoxP3^+^ [5, 14, 15]. RA imprints gut-homing molecules on Tregs, so they can return to LP and proliferate in an IL-10 dependent mechanism [16]. iTregFoxP3^+^ cells induces the secretion of IL-27 by DCs CD11b^+^population in LP, enhancing the production of IL-10 by type I regulatory T cells (Tr1) [17, 18]. Regulatory cells and immunosuppressive cytokines present in the intestinal LP and mLN promote tolerance over inflammation in the GALT, on systems homeostasis. A complex network representation of the oral tolerance can be seen in the graph of Fig 1. From these assembling, we devise a generic model which shall heuristically produce a way to study the oral tolerance considering it as a complex stochastic phenomenon.

### The dynamical stochastic model

A stochastic dynamical system is subject to the effect of noise or some sort of fluctuation through time. We classify a fluctuation as stochastic when it is conceived by a system with large number of variables or a high degree of freedom [19]. One of the most fundamental studies in this area was realized by Einstein as he was trying to understand how suspended particles moves in a random fashion, named Brownian movement [20]. The equations of motion of these systems can be approached both analytically and numerically depending on its configurations. The use Markovian Chain is often useful in order to predict and model stochastic systems [19]. If we look at the cross literature between stochastic systems and immunology, we can see that this modeling is often. There is a number of studies in which the stochastic dynamics is applied to model the dynamics of immune systems, for example: the modeling of the escape rate in HIV/SIV infections [21]; the modeling of the viral kinetics under immune control also during HIV infections [22]; the modeling for the stochastic behavior of cytotoxic T cell responses [23]; the strategies of viral spread and reproduction as a stochastic dynamical system [24]; the modeling of early viral infections using stochastic theory [25]; the general way that viruses reproduction responses in a dynamical fashion [26]. As this list could go much further, we are instigated in modeling our phenomenon of interest in the same manner.

To understand how, in a statistical sense, interactive immunological components of the oral tolerance are related to each other, we propose a new model based on a variation of random walkers in our network model and its asymptotic behavior. Overall, this process aims to generate statistical quantities that give us a notion on how oral tolerance phenomenon occurs via complex behavior between its immunological components (i. e. cells and molecular components involved into the immunological process). In other words, we want to generate a quantity to assess the diffusion of stimulation throughout the network.

The interactive network was considered as a fixed network, with constant number of vertices and edges. Note that the network is complete (i. e. there is no unconnected vertices on the graph). The connectivity between vertices can be represented by an adjacency matrix; if there is a directed edge connecting the vertex *i* and *j*, the value of the adjacency matrix’s element *a*_*ij*_ will be 1 and 0 otherwise.

Aiming to understand the resulting product between immune components and their interactions, we will focus on the distribution of out degree, *k*_*out*_. Consider a generic vertex *i*, its out degree can be written as

$$k_{i\;out}\;=\;\sum_{j}a_{ij},$$ (1)

which is the sum of the elements of the row of matrix, *a*_*ij*_. Graphically, this quantity is expressed into the graph as the number of edges pointing out from the vertex *i*.

As these two topological concepts are established, we can define the random walk on the network for the purposes of our study. In general, the random walk on a complex network can be understood as a stochastic process in discrete time-steps, in which a walker follows a path determined by the network’s topology, considering his actual position into it. In our model, walkers depart from a fixed vertex, the source (the antigen vertex). To formalize this process, some rules of “walking onto the network” can be summarized:

1. On an initial time *t*, a generic walker *w*, initially located at the vertex *i* of the network, changes his position to a vertex *j* as the times changes from *t* to *t+1*;
2. The walker is allowed to shift positions, from a vertex *i* to any other vertex *j* if and only if *j* is a first degree neighbor of *i* (i. e. separated only by one edge); given the time changes of *t* to *t+1*. The probability of shifting is given by *p* = 1/Σ_*j*_ *a*_*ij*_, the formalism of these probabilities are developed in the next section;
3. Additionally, the walker must respect the directionality of the edges; if the walk is found on the vertex *i*, he will only go to another vertex *j* if there is a edge point from the vertex *i* to the vertex *j*;

When we introduce more walkers and each walker has its position and dynamics, it is of convenience to create a positional walking vector *W*(*g*;*t*). Given an initial time *t*, this vector of size *N*(*t*) (this function stands for the total number of walkers on the network in the time *t*), and its components are associated to a walker on the network:

$$W\left(g;t\right)\;=\;\left(w_{1}(t),\;w_{2}(t),\;w_{3}(t),\;\ldots w_{N(t)}(t)\right),$$ (2)

where *g* stands for the graph which the walker is found, and the generic position *w*_*n*_(*t*)∈ 1,…,33 is the node position of the n-esimal walker inserted onto the network. The probability of position change of a walker to go from *i* to *j* provides the frequency with which the interactions occur in our immunological network. Since the network is directed, then the probability for a walker to arrive on the vertex *j* from the vertex *i* is usually distinct from arriving to *i* from *j*, since the out degree of *i* and *j* is typically different. To infer about the concentration of the immune components, we introduce the concept of positional topological vector where components provide the number of walkers at a given time *t* at a given vertex

$$S\left(g;t\right)\;=\;\left(\sigma_{1}\left(t\right),\;\sigma_{2}\left(t\right),\;\sigma_{3}(t)\ldots \sigma_{V}(t)\right),$$ (3)

where *σ*_*i*_(*t*) stands for the number of walkers in the time *t* on the vertex *i*, in a network with *V* = 33 vertices. We can perceive that for each time-step, there will be a contribution of walkers associated to the topological position (vertex). So, if we calculate the relative value of each component’s value of the above-stated vector *S*, we can write a quantity associated to each vertex that we call flux of walkers:

$$f_{i}\left(t\right)\;=\;\frac{\sigma_{i}(t)}{N(t)},$$ (4)

with *i* = 1,…33. We emphasize *N*(*t*) = *t*, since we consider that at each iteration a new walker enters the network, then the flux vector is defined as

$$F\left(g;t\right)\;=\;\left(f_{1}\left(t\right),\;f_{2}\left(t\right),\;f_{3}\left(t\right),\;\ldots \;f_{n}\left(t\right)\right).$$ (5)

Higher values of *f*_*i*_(*t*), means that the vertex *i* is being more activated in an immunological sense, and it can be also be understood that more information is passing through this agent. This vector is of statistical interest to this work, for it is a dynamical quantity that depends on the random choice of each walker on the vertices of the network. Since the network is connected, and as demonstrated in the following, as *t*→∞, the flux vector *F*(*g*,*t*) tends to a stationary distribution *F*(*g*,*t*→∞).

To understand how topological changes in the network graph (*i*. *e*., a KO) causes changes in the statistical behaviour of the walker, we study the quantity

$$F\left(g;t\right)-F\left(g^{'},\;t\right)\;=\;\Delta f.$$ (6)

Where *F*(*g*′,*t*) represents the flux vector for a KO network *g’* and *Δf* the difference flow vector with components Δf_1_,Δf_2_,…,Δf_n_. The values of Δf_i_ can be negative or positive: if positive, it means that the activation of the agent is increased by the KO; but negative, it means that the activation, overall, is decreased by the KO. In general, these differences may be negative or positive, given that *f’* can be higher than *f*, or vice-versa. For each of these cases, we define the relative error by

pi = Δfi(t)fit, for Δfi>0.Δfi(t)fi't, for Δfi<0. (7)

which scale the variation Δf_i_ with respect to the largest density, either *f*_*i*_ or *f*_*i*_′. We also define the average of the relative error by

$$P\left(g;g^{'};t\to \infty \right)\;=\;\frac{\sum_{i\;=\;1}^{n}p_{i}}{n}.$$ (8)

Where values close to zero means that the topological modification (*g*→*g*′) caused no significant impact to the dynamical behaviour of the network. When values are close to one, it indicates that the topological modification caused a major modification to the dynamics concerning its normal functioning the distribution given by *F*(*g*;*t*→∞).

Our quantities rely upon how we understand the oral tolerance; we propose that the information unchained by the presence of innocuous antigens on the intestinal lumen starts information processing that stimulates all local immunological components and states the immunological network model. This define what we understand, topologically and dynamically, as oral tolerance in terms of the model; from this point on, we can study how each component behave and how it is important for the whole phenomenon throughout the KOs.

The central quantity from our model is the components associated to the vector *F*(*g*,*t*), which are the values of *f*_*i*_(*t*); these are a distribution of relative number of walkers. This distribution is the parameter of determination of how much stimulus (i. e. number of walkers on vertices) each immunological component must receive in order to define the studied phenomenon. One may wonder how this model can provide previsions about biological insights; we let this part to the discussion latter section.

### Algebraic development of the model

Aiming to seek an analytic way to unravel the values of *f*_*i*_(*t*), we introduce the transition matrix *T* of a graph. This matrix is composed by elements which corresponds to the probability of a walker to leave the vertex *i* and arrive at vertex *j* defined as

$$t_{ij}\;=\;\frac{1}{k_{i\;out}},\;$$ (9)

in a general way, each values of *t*_*ij*_ will compose the stochastic matrix *T*. For our interest, we wish to explore the following expression:

$$f_{i}\left(t+1\right)\;=\;Tf_{i}\left(t\right),$$ (10)

which implies,

$$f_{i}\left(t\to \infty \right)\;=\;T^{\infty }f_{i}\left(t\;=\;0\right).$$ (11)

Interestingly, the rows of *T* ^∞^ represents the asymptotic values of *F*(*g*;*t*→∞),which are stationary values for the proceeding vector. This approximation allows us to obtain instantaneous values analytically which should, for instance, be achieved via iterative methods as explored on the next topic of this paper.

Since the transition of states given by the elements of T follows a Markovian process, we consider {*λ*_*i*_} the eigenvalues of *T* to following the condition |*λ*_1_|≥|*λ*_2_|≥…|*λ*_*n*_|. Since the Markov chain is ergodic, the system arrives on a stationary distribution $T^{\infty }\equiv \pi \;=\;\left(\begin{array}{c} \vec{v_{r}}\\ \vec{v_{r}}\\ \vec{v_{r}} \end{array}\right)$, for π∈ℝ^33×33^ featuring the Perron Frobenius theory for nonnegative matrices [27], if |*λ*_1_| = 1 and |*λ*_*i*_|<1 for all 2≤*i*≤*n* where $\vec{v_{r}}$ represents the normalized eigenvector of the unitary eigenvalue; we also can affirm that if the eigenvalues are real, the Markov chain is reversible. We calculated the eigenvalues of the immunological network and we found {|*λ*_1_| = 1; |*λ*_2_| = 0.958; |*λ*_1_| = 0.741; |*λ*_1_| = 0.718;…}. This satisfies the above stated feature in order to apply the Perron Frobenius nonnegative theory to our case. Meaning that $F\left(g;t\to \infty \right)\;=\;\vec{v_{1}}$. Considering this analytical approach, Eq (8) is used considering that $\Delta f_{i}\;=\;\vec{v_{1}}\left(g\right)-\vec{v_{1}}(g^{'})$.

### Algorithm development and statistics

In here, we describe the details of the algorithm that incorporates all steps described on the model and has the goal of generating a mean flux vectors by some fixed random walking parameters. The main parameter is the time *t* = 10^4^ for walk, reminding that at each time-step, it will be created a walker, the positional walking vector receives a new component with value 1, which means that the created walker is “spawned” on the node1 in the network. Having a walker placed in node 1 represents, for our immunological network, the presence of a stimulatory antigen in intestinal lumen.

The other parameter of the algorithm is the number of realizations *L* that all this process is repeated. The many realization (*L* = 10^4^) are necessary in order to calculate an average value of the flux vector that approaches quickly the expected values obtained via Eq (5). The mean value of the flux vector is calculated by:

$$\overset{-}{F}\left(g,\;t\;=\;10^{4},\;L\;=\;10^{4}\right)\;=\;\frac{\sum_{L\;=\;1}^{L}F(g;t)}{L}.$$ (12)

So for an initial graph *g*, we calculated the $\overset{-}{F}\left(g;t\;=\;10^{4};L\;=\;10^{4}\right)$ vector, at time *t* = 10^4^. To do statistics considering the KO networks, we do the previous analysis to networks, constructing by removing one vertex. A KO graph is a graph constructed by the removal of a vertex of the original graph; normally the knockout graph takes the name of the vertex removed from it. As the original “healthy” graph consists of 33 vertices, or 32 knockable vertices (given that the antigen vertex has no meaning for KO), we studied how this removal influenced the mean flux vector for each case.

Given these premises, we can compare the values of the mean flux vector of the standard graph with other KO graphs mean flux vector, by calculating the mean relative deviation

$$\overset{-}{P}\left(g;g^{'};t\;=\;10^{4}\right)\;=\;\frac{\sum_{i\;=\;1}^{V}{\overset{-}{p}}_{i}}{V},\;$$ (13)

where *g* is the normal graph and *g’* stands for the KO graph. The value of $\overset{-}{P}$ tell us how the KO was significant for the oral tolerance process in relation to *L* simulation of the specified random walk.

## Results

**Fig 2:**
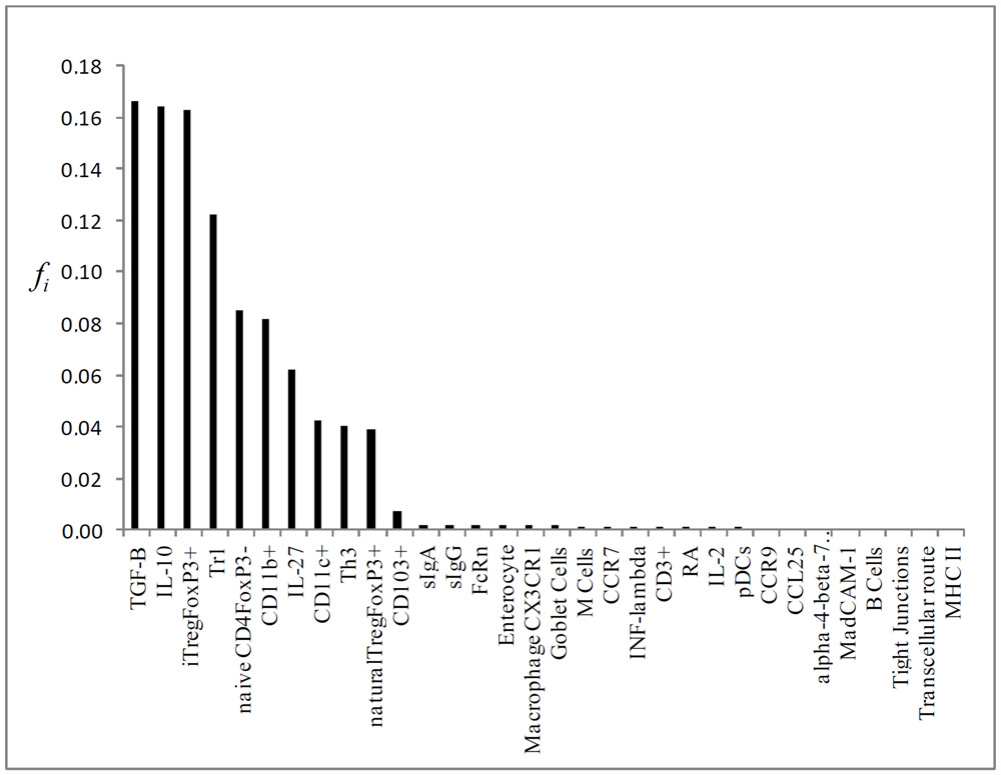
Graphical representation of the flux of walkers f(i) of the mean flux as a histogram.

**Fig 3:**
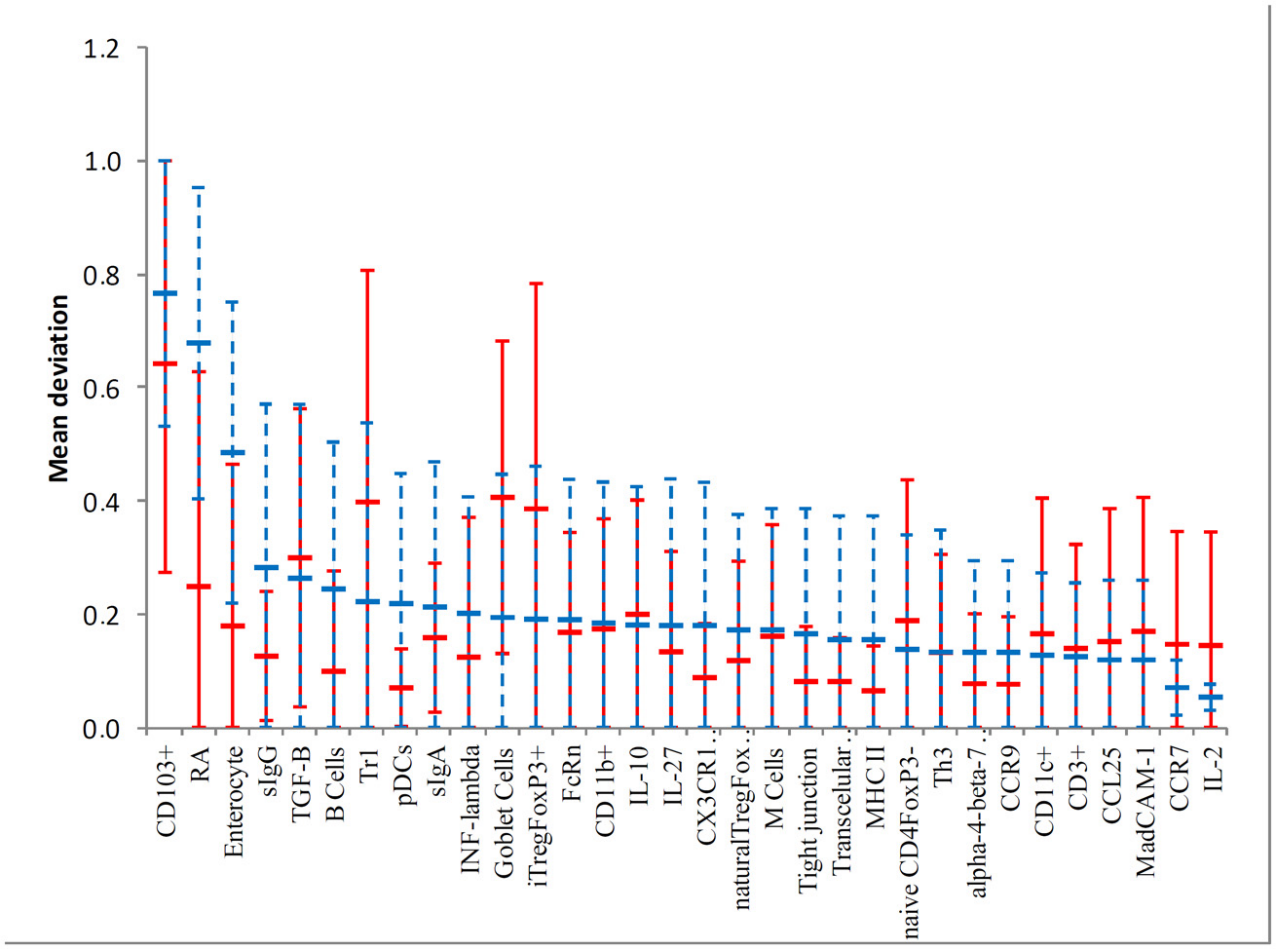
Graphical representation of the mean deviation. Each KO calculated analytically (blue dashed lines) and via simulation (*t* = 10^4^;*L* = 10^4^) of random walk (red lines).

Running the stochastic model using the system’s parameter for *t* = 10^4^ and *L* = 10^4^, we calculated the standard flux vector (*i*. *e*., healthy network) which is illustrated in Fig 2. The mean relative deviation for the analytic and simulation approach can be found on Fig 3; it depicts the relatives flux for each knock-out vertex in relation to the standard flux. Table 1 displays the values. Interestingly, the knockout of CD103^+^ vertex caused the greatest impact on network standard flux for even both the analytical and simulation approaches, resulting in a mean relative deviation of 0.64 and 0.76, respectively. Following CD103+ DCs KO, we found iTregFoxP3^+^ KO, and Tr1 KO leading to a mean relative error of 0.37 and 0.38 respectively, and for TGF-β KO and RA KO, 0.29 and 0.23 respectively. The results are in touch with biological expectation of the model, where DCs CD103^+^ plays critical roles in the generation of Tregs, through mechanisms dependent on TGF-β and RA. Tr1 cells acts by the secretion of IL-10, helping to maintain tolerance to commensal antigens [5, 14, 28, 29].

**Table 1:** Immunological components along with their values of mean deviation impact in relation to the healthy graph.

Immunological component	Analytic approach	Random walk approach
**CD103+**	0,765799618	0,641861422
**RA**	0,678317118	0,24839991
**Enterocyte**	0,484672223	0,178777324
**sIgG**	0,282401698	0,125713213
**TGF-B**	0,263407182	0,299158934
**B Cells**	0,244445065	0,098758938
**Tr1**	0,221685375	0,397771101
**pDCs**	0,218277464	0,069850561
**sIgA**	0,212605704	0,158329195
**INF-lambda**	0,201161196	0,123635554
**Goblet Cells**	0,193837326	0,406126091
**iTregFoxP3+**	0,190969942	0,38559257
**FcRn**	0,190124221	0,16774982
**CD11b+**	0,183867254	0,173765649
**IL-10**	0,180737501	0,19962195
**IL-27**	0,179652767	0,133529241
**CX3CR1 Macrages**	0,179451274	0,08745167
**naturalTregFoxP3+**	0,172121275	0,117944767
**M Cells**	0,172117375	0,160550102
**Tight junction**	0,165135013	0,080944792
**Transcelular route**	0,154885332	0,080618764
**MHC II**	0,154885332	0,064364371
**naive CD4FoxP3-**	0,137615325	0,188077441
**Th3**	0,132918693	0,132295778
**alpha-4-beta-7 integrin**	0,132513876	0,077010842
**CCR9**	0,132513876	0,076179264
**CD11c+**	0,127130694	0,165421145
**CD3+**	0,124570624	0,139382369
**CCL25**	0,119132158	0,151484592
**MadCAM-1**	0,119132158	0,169699051
**CCR7**	0,070059124	0,146692611
**IL-2**	0,05306735	0,14444037

The values can be interpreted as the topological impact caused on the network for the analytic method and random walk simulation.

To compare both approach of finding the KOs impacts, we calculated the relative error averaging the differences of same KOs between analytic and simulation values. We found that percentage difference between the simulation and analytical method is about 8% with parameters *t* = 10^4^ and *L* = 10^4^. So we retested them for lower values for these parameter (*t* = 10^3^;*L* = 10^4^) and the error was about 9,1%. We assumed that the overall tendency of the simulation results the analytical results are to converge on a limit value for the parameter *t* and *L*. However, to do so, we would need an infinite computational power in order to calculate this difference precisely. As this difference played not an utmost role into the purpose of our work, we didn’t go deeper into this aspect.

**Fig 4:**
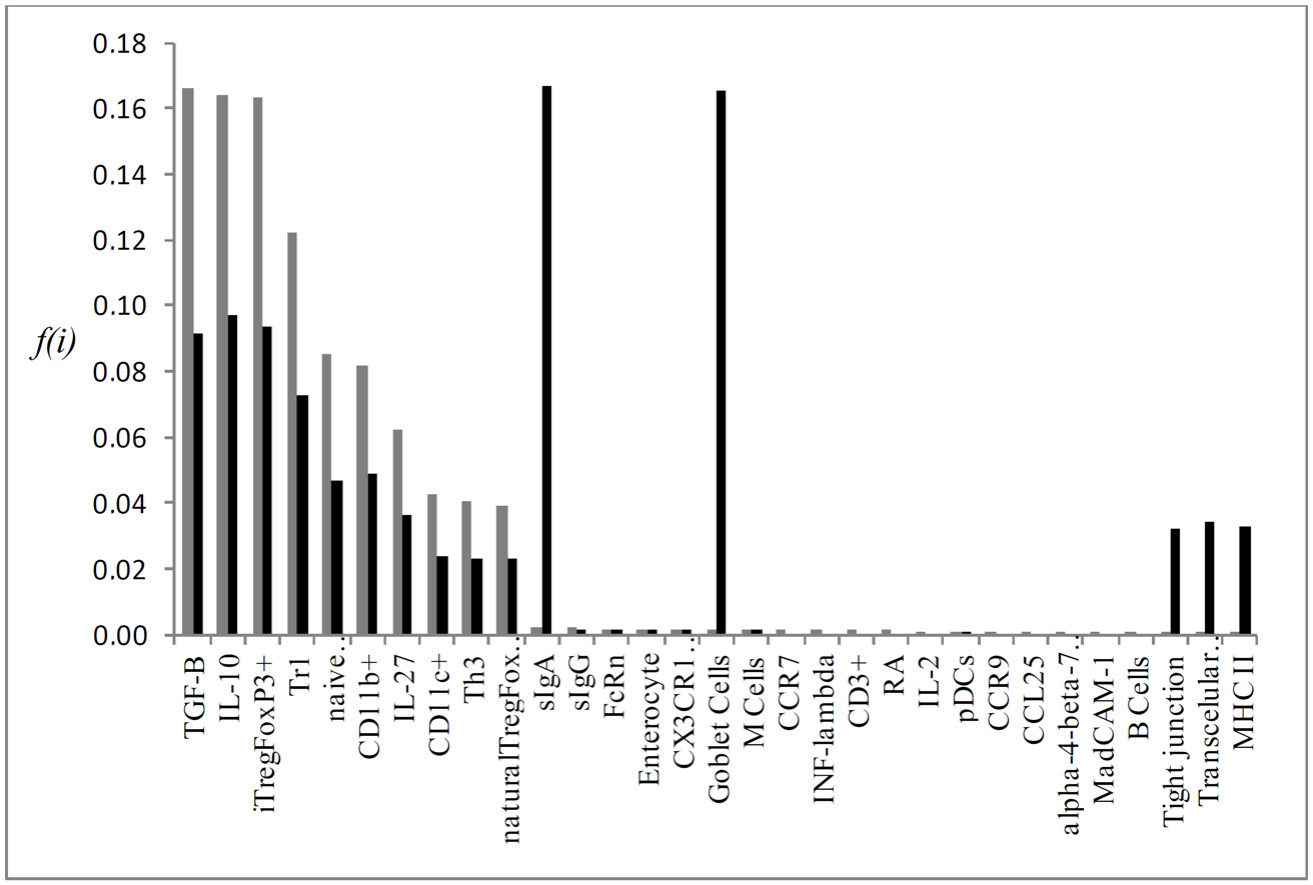
Graphical representation of the mean flux of walkers f(i) (black histogram) and the KO CD103^+^ (grey histogram).

**Fig 5:**
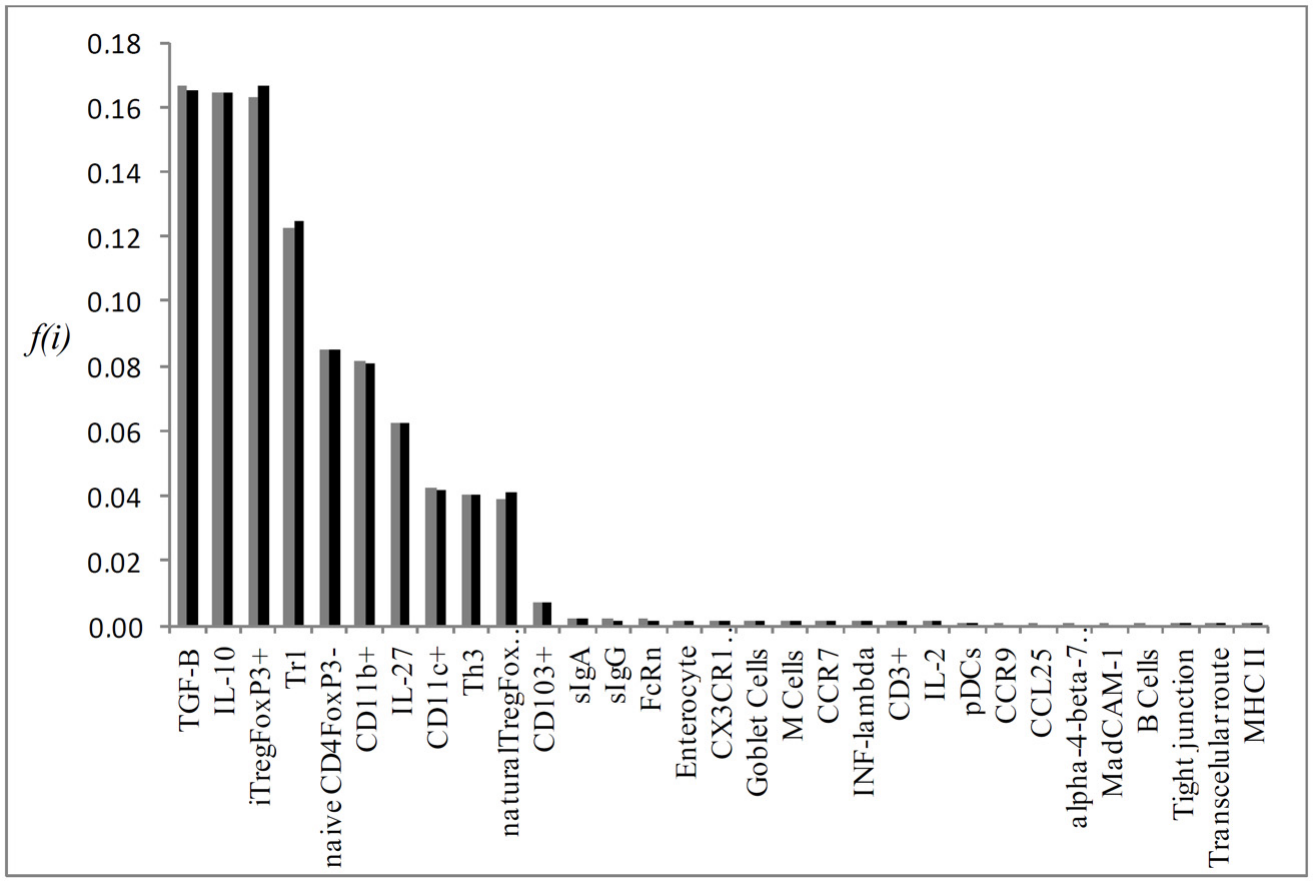
Graphical representation of the mean flux of walkers f(i) (black histogram) and the KO RA (grey histogram).

**Fig 6:**
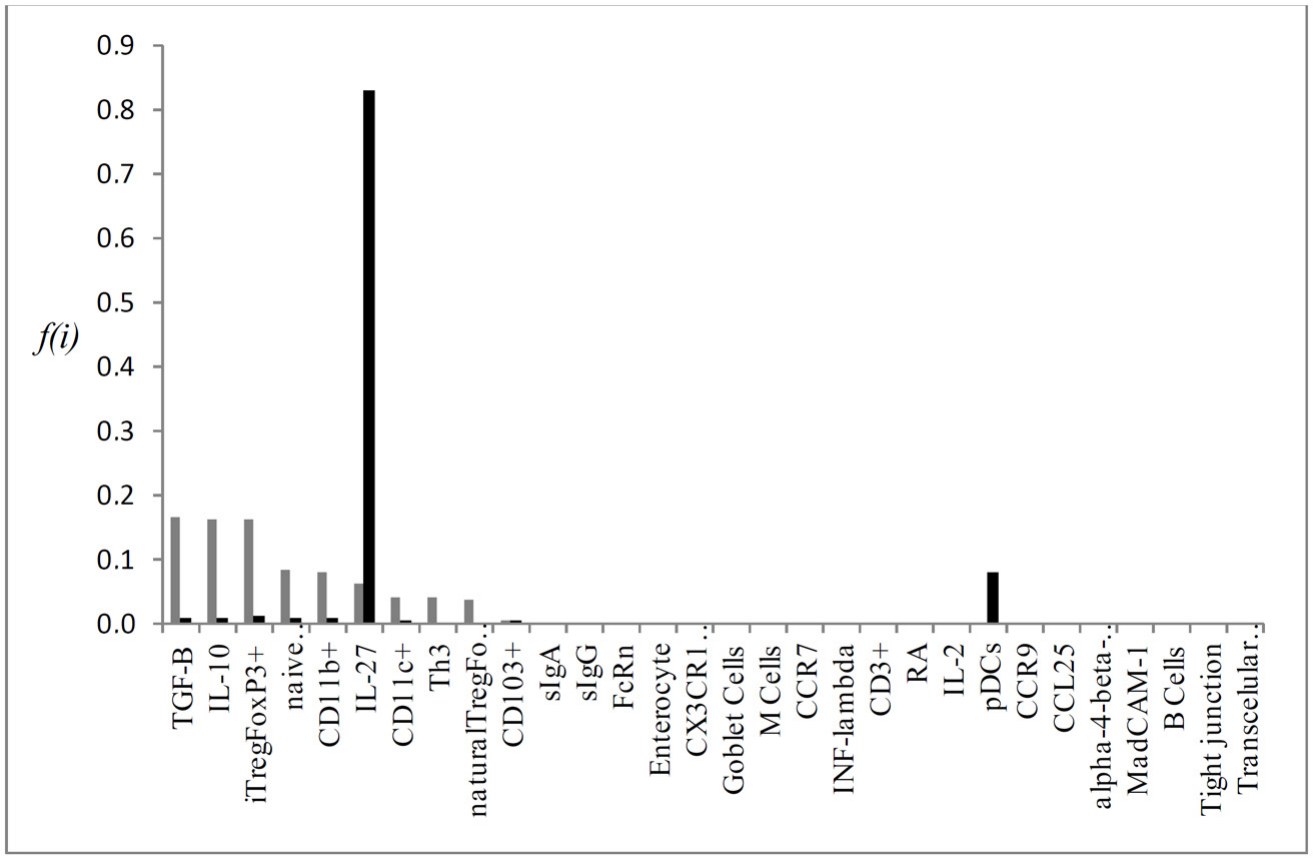
Graphical representation of the mean flux of walkers f(i) (black histogram) and the KO Tr1 (grey histogram).

**Fig 7:**
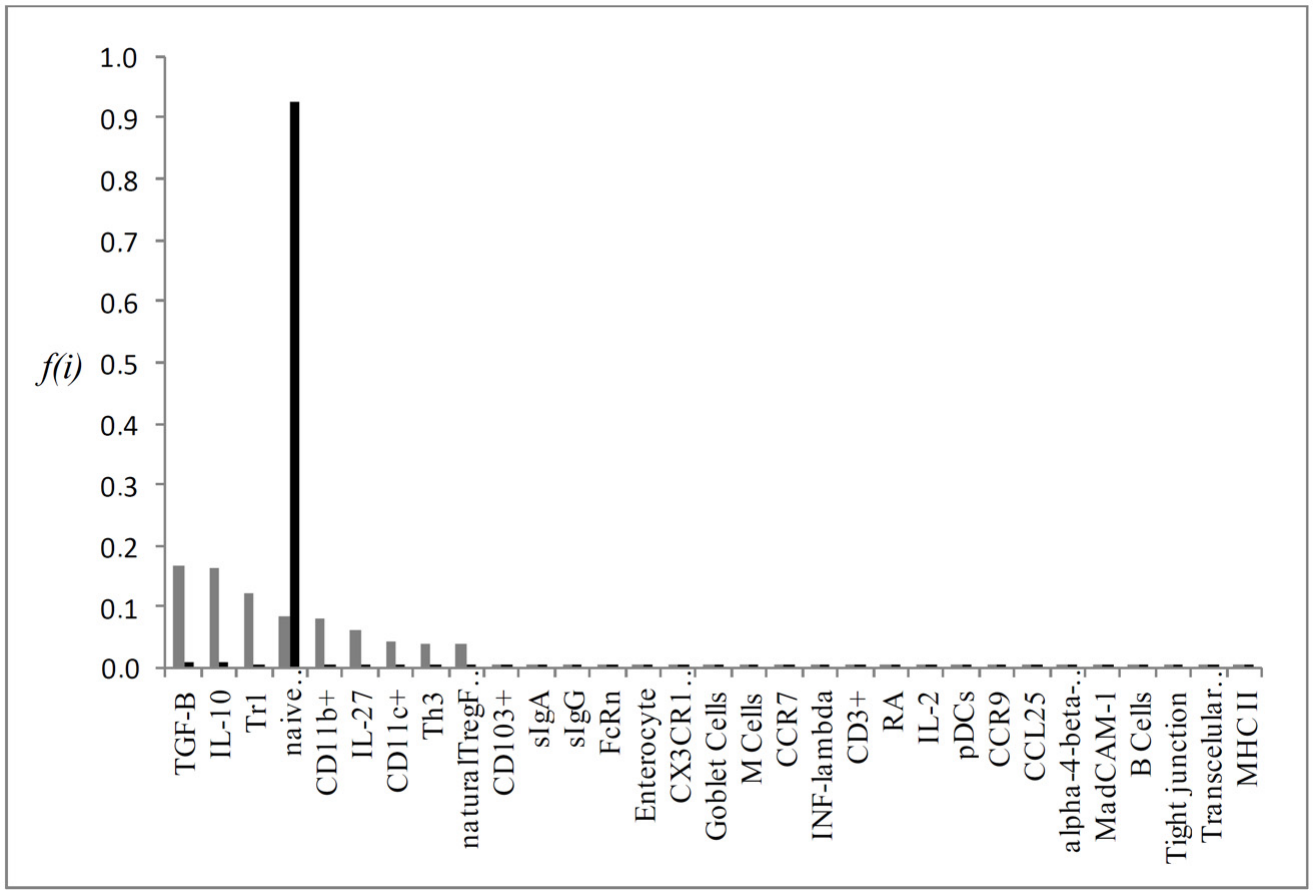
Graphical representation of the mean flux of walkers f(i) (black histogram) and the KO iTREGFOXP3^+^ (grey histogram).

**Fig 8:**
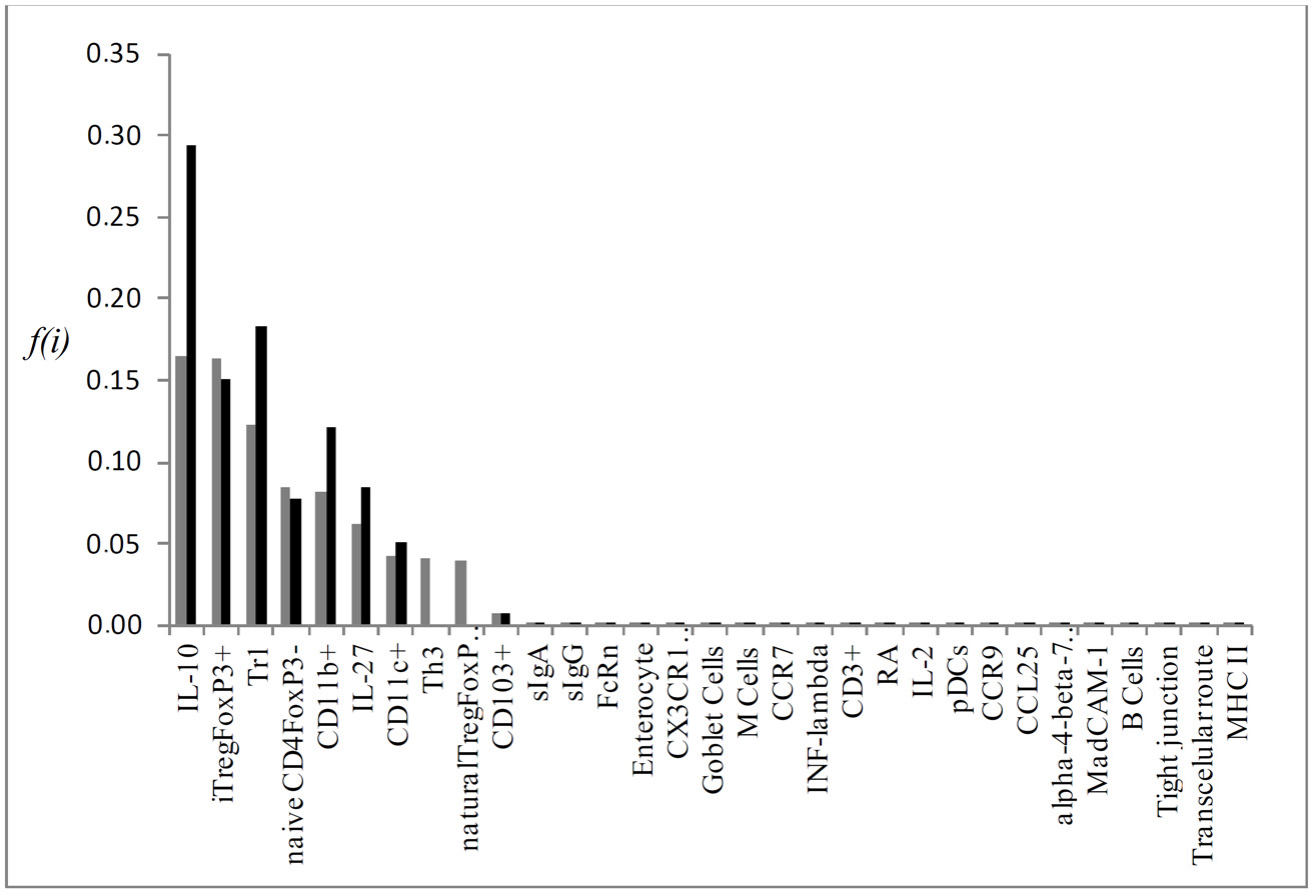
Graphical representation of the mean flux of walkers f(i) (black histogram) and the KO TGF-β (grey histogram).

Individual flux of the most important KOs $\left(\overset{-}{P}>0,25\right)$ for the simulation results, which are: CD103^+^, RA, Tr1, iTregFoxP3^+^ and TGF-β (Figs 4, 5, 6, 7 and 8, respectively). A discussion of the biological implication of each of these KOs is made afterwards.

## Discussion

### Implications and interpretation of the model

Around 40 years ago, Niels Kaj Jerne proposed a new theory to explain the basis of the immune system behavior, suggesting the existence of a functional network, centered on patterns of idiotype recognition, carried by lymphocytes [30]. This brief concept was the spark that awakened interest in researchers to view the nervous system not only as a network of physical interactions but also as a network of functional interactions. Notable properties of the immune system as: diversity, self-regulation, memory, connectivity and stability to perturbations, lead to the construction of mathematical models based on differential equations, automaton cellular model and Boolean functions [31–34].

One of the most intriguing properties of the immune system is self-regulation; the development of limited responses when constantly perturbed. In this scenario, gut represents the site where most of antigenic contact occurs in the organism. The adult human gut has nearly 500 to 1000 microorganisms’ species, in a density of 10^14^ cells, corresponding to ten times more the number of somatic human cells and surpassing by two orders the human genetic potential [35]. Beyond the gut microbiota, 130 to 190 g of food proteins are daily absorbed, consisting as another major source of antigenic stimulation in the GALT [36]. However, when in physiological conditions, the prevalent response in the GALT is tolerance. The phenomenon occurs through the multiple action of immune components (dendritic cells, T cells, plamocytes, cytokines) interacting in a unique microenvironment, that is shared by other mucosal sites [3, 4, 7].

In the present work, we investigated the immune components and respective interactions that generate, maintain and regulate the oral tolerance, through the creation of a complex network of interactions. The majority of biological networks are scale-free, displaying the degree distribution of vertex in a power law [37]. In our network, the low clustering coefficient and degree distribution of vertex don’t display power law dependence, so there is no characterization as a scale-free network. Although the topology didn’t show any relevant feature, we assumed that our network was too little to the expected feature to be displayed. As the phenomenon of our study is limited to the oral tolerance, we considered a small subset of the total immunological system, so for topological analysis our network is insufficient. Zhao *et al*. (2013) [38] obtained a network of interactions between immune genes activated in the spleen during *Haemophilus parasuis* infection in porcine. Although the size of the network satisfied the conditions for analyzing topological properties, the network could not be considered as a small world even though it was scale-free.

Regarding our stochastic model, the results obtained in the standard flux suggest that iTregFoxP3^+^ cells are constantly activated upon antigen presentation, noting that TGF-β and IL-10 are the two main suppressor cytokines secreted by these cells. Induced Tregs in peripheral tissues play its suppressor role (inhibition of Th1, Th2 and Th17) in a cytokine dependent mechanism, while natural Tregs act preferentially in a cell-cell manner [39]. The high activation in naïveCD4FoxP3^-^ cells in the model, corroborates its expected role: renewal of T cells in the intestinal lamina propria and mesenteric lymph nodes. With the constant entry of antigens (food proteins, microbiota, other environment antigens) in the intestinal lumen, new Treg cells are generated (FoxP3^+^, Tr1, Th3) and even non-regulatory T cell subsets, like Th17 cells, maintains the GALT homeostasis [40–41]. Activated TregFoxP3^+^ cells induce the secretion of IL-27 by dendritic cells, which stimulate the differentiation and proliferation of Tr1 cells and production of IL-10 [42]. This mechanism acts as a positively regulator of IL-10 in LP and mLN [13]. The other two main phenotypes of dendritic cells present in the GALT (CD11b+ CD103- and CD11c+ CD11b- and in the liver (plasmacytoid dendritic cells—pDCs) are found in the standard flux. Dendritic cells are essential in the process of oral tolerance, as well as in the immune response to harmful antigens and pathogens. CD11b^+^ cells produce IL-10, and consistently maintain the Treg population in LP. CD11b^+^ are also important in the processing of antigens in Peyer’s patch [43, 44]. At last, thymus derived regulatory T cells (nTregs) areactivated in the standard flux, maintaining tolerance to self-antigens. Pacholczyk *et al*. (2007) [45] showed that nTregs can cognate non-self antigens, tolerating in this way, commensal antigens. Although they might be dispensable in the induction of oral tolerance [46], the majority of nTregs respond to commensal antigens in the GALT [47]. Briefly, the standard flux found in our model displays some remarkable features of oral tolerance; the activation of Treg cells (iTregFoxP3^+^, Tr1, Th3, nTreg), dendritic cells (DCs) and production of suppressive cytokines TGF-β and IL-10, which occurs by different cell types present in LP (i. e. DCs, Tregs, Macrophages, Enterocyte) [3].

### Analysis of KOs and its biological interpretation

As the network vertices were individually removed (knockout) and relative deviations were calculated, we quantify the relative importance of all particular immunological elements involved on the process. Knocking out the vertex CD103^+^ caused the greatest impact on network dynamics, as observed in the analytic and interactive model. DCs CD103^+^ are the main population of dendritic cells in intestinal LP and mLN. The expression of αEβ7 integrin restricts the migration of these cells between LP and mLN, which corresponds to its role in receiving antigens from intestinal lumen and load these antigens to mLN, where they are presented to *naïve*CD4^+^ T cells [13]. DCs CD103^+^ produce suppressor cytokines TGF-β, IL-10 and retinoic acid (RA) (Fig 3), through the expression of RALDH2, essential for the generation of iTregs. Beyond its synergic action with TGF-β in the generation of iTregs, RA is also important in the synthesis of gut-homing molecules, like the α4β7 integrin and CCR9, allowing Tregs to return from mesenteric lymph nodes to intestinal LP [4, 48]. Mice knockout for CD103^+^ (CD103^-/-^) showed lower frequency of Tregs and T effectors, significant reduction in the expression of gut-homing molecules in T cells, and oral tolerance induction was impaired [49]. Moreover, the deficiency of p38α in CD103^+^ inhibit the generation of induced Treg while promoting Th1 cell development [50] As observed in our model, the absence of DCs CD103^+^ (Fig 3) lead to a significant reduction in the activation of vertices corresponding to suppressive cytokines (TGF-β, IL-10 and IL-27), reduction on all regulatory T cells (FoxP3^+^, Tr1, Th3, thymus derived Tregs) and on other DCs subsets (CD11b^+^ and CD11c^+^). The high flux of information of vertices like Goblet Cells, Tight junctions and sIgA reflects the accumulation of antigen on these routes, where primarily CD103^+^ are responsible for capturing and loading antigens for T cell priming. However, it is not expected that in the absence of CD103^+^, levels of sIgA or frequency of cells like goblet cells might be increased in biological system. The increased activation of plasmacytoid dendritic cells (pDCs) in the CD103 interactive and analytic knockout may show that other dendritic cell populations, as pDCs that promote oral tolerance in the liver, can account as another source of antigen presentation in the absence of CD103^+^ DCs. pDCs are essential for oral tolerance induction and maintenance as observed in mice model [51].

The removal of iTregFoxP3^+^ and Tr1 resulted in a relative error of 0.37 and 0.38 respectively. FoxP3 is the key regulator of Tregs, being indispensable for its function [52]. Mice null FoxP3^-/-^ developed devastating autoimmune disease (Fig 6), marked by splenomegaly, lymphadenopathy, insulitis, severe skin inflammation, delayed body development and less survival [44, 45]. This data does not discern between inducible Tregs and natural Tregs, developed in the thymus. However, evidence indicates that iTregs are the main responsible for oral tolerance [53].

The lack of specific markers for Tr1 cells make it hard to comprehend its specific functions and use in clinic. Recently these cells were identified as CD4^+^CD49b^+^LAG-3^+^. Tr1 cells are also induced in the GALT, and respond through the high production of IL-10 [54]. As the knockout of FoxP3, mice that lack LAG-3 exhibit leucocyte infiltrate in multiple organs followed by autoimmune disease [55]. The pronounced increase in IL-27 in our model when Tr1 (Fig 5) was removed is due to the induction mechanism that IL-27 secreted by dendritic cells (pDCs) does on differentiation and proliferation of Tr1 cells. However, the main cytokine secreted by Tr1 cells, IL-10, is dispensable for oral tolerance induction in low doses. Whereas the encephalomyelitis was worse in IL-10^-/-^ mice, the oral administration of myelin glycoprotein of oligodendrocytes (MOG _35–55_) result in improvement of disease in all groups [56]. TGF-β plays distinct functions in the GALT (Fig 7); promotes the expression of FoxP3, regulates Treg function, as well as the polarization of Th17 under the presence of IL-6. As FoxP3 and LAG-3 null mice, mice that lack TGF-β present spontaneous autoimmune disease, and depletion of TGF-βR II of T cells resulted in a similar phenotype, but less aggressive, marked by spontaneous activation of T cells, production of autoantibodies and leukocyte infiltrate in multiple organs [57]. It is worth noting that mice null for TGF-β do not provide a clear view about TGF-β and its function on oral tolerance, since TGF-β takes part in numerous processes linked to organism development, and in its absence, drastic changes occur [58]. In the lack of TGF-β receptors in T cells, less CD103^+^ cells developed and INF-γ levels were increased [59]. TGF-β and IL-10 supports tolerance in the GALT. When TGF-β was removed from the network (Fig 7), an increase in IL-10 activation and so Tr1 cell differentiation may act like a counter mechanism so to maintain tolerance in the GALT. iTregs levels were a little lower and Th3 levels were completely abrogated, as TGF-β is essential for Th3 development. Karlsson *et al* (2010) [60] demonstrated that in the absence of secretory antibodies (IgG and IgA) in the gut, oral tolerance could normally be induced. As the mucous barrier becomes leakier, it is possible that more microorganisms and food antigens access intestinal LP. The main feature observed in the sIgA interactive knockout was an increase in retinoic acid (RA) and CD103^+^ cells activation. Although there is no data concerning RA levels in secretory Ig knockout mice, the increased activation of CD103^+^ DCs might be due to an increased sampling of lumen antigen as the intestinal barrier is partially disrupted [61].

## Conclusion

The model based on complex network is capable of describing the dynamics of the immune system in oral tolerance in phenomenological terms. Our model addresses both topological properties and dynamical relations, implemented by a random walk algorithm. The major limitation found in the model is that it lacks a fully quantitative component. Nevertheless, the construction of a qualitative stochastic model for oral tolerance could reflect empirical implications, through the standard flux results and relative error based on individual knockouts. Our model, allied to other models [62, 63] stand as a starting point in the construction of quantitative network models that may describe the kinetics and intensity of casual relationships between the components of the immune system.
